# A Master-Slave Surveillance System to Acquire Panoramic and Multiscale Videos

**DOI:** 10.1155/2014/491549

**Published:** 2014-03-03

**Authors:** Yu Liu, Shiming Lai, Chenglin Zuo, Hao Shi, Maojun Zhang

**Affiliations:** Department of System Engineering, College of Information System and Management, National University of Defense Technology, Changsha 410073, China

## Abstract

This paper describes a master-slave visual surveillance system that uses stationary-dynamic camera assemblies to achieve wide field of view and selective focus of interest. In this system, the fish-eye panoramic camera is capable of monitoring a large area, and the PTZ dome camera has high mobility and zoom ability. In order to achieve the precise interaction, preprocessing spatial calibration between these two cameras is required. This paper introduces a novel calibration approach to automatically calculate a transformation matrix model between two coordinate systems by matching feature points. In addition, a distortion correction method based on Midpoint Circle Algorithm is proposed to handle obvious horizontal distortion in the captured panoramic image. Experimental results using realistic scenes have demonstrated the efficiency and applicability of the system with real-time surveillance.

## 1. Introduction

**Figure 1:**
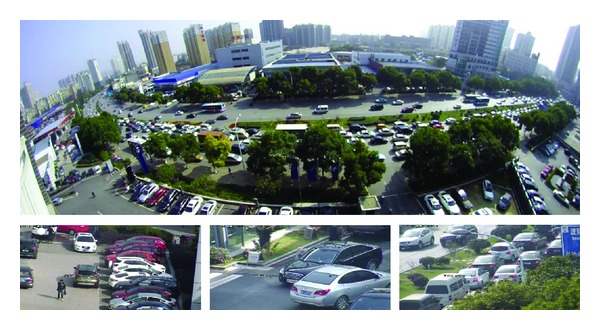
The fish-eye panoramic image captured by the master camera and zoom-in image captured by slave camera with distant details.

Digital video surveillance has become commonly used in public and private places such as government buildings, military bases, car parks, and banks, and so forth. Traditional monitoring cameras can only cover a limited area, leading to “blind spots.” Developments in panoramic imaging technology offer significant advantages over traditional surveillance systems. They can monitor an area that covers 180° or 360° and so replace several traditional cameras. Nevertheless, images captured by panoramic cameras have limited range of scale due to the relatively reduced resolution. Comparatively PTZ dome cameras can focus on areas of interest rapidly by decreasing or increasing focal length. The master-slave camera composed of a fish-eye panoramic camera and PTZ dome camera combines the advantages of both (Figure 1). The fish-eye panoramic camera is responsible for acquiring global and wide images in the large surveillance area, and the PTZ dome camera is used to acquire multiscale videos for more detailed information.

**Figure 2:**
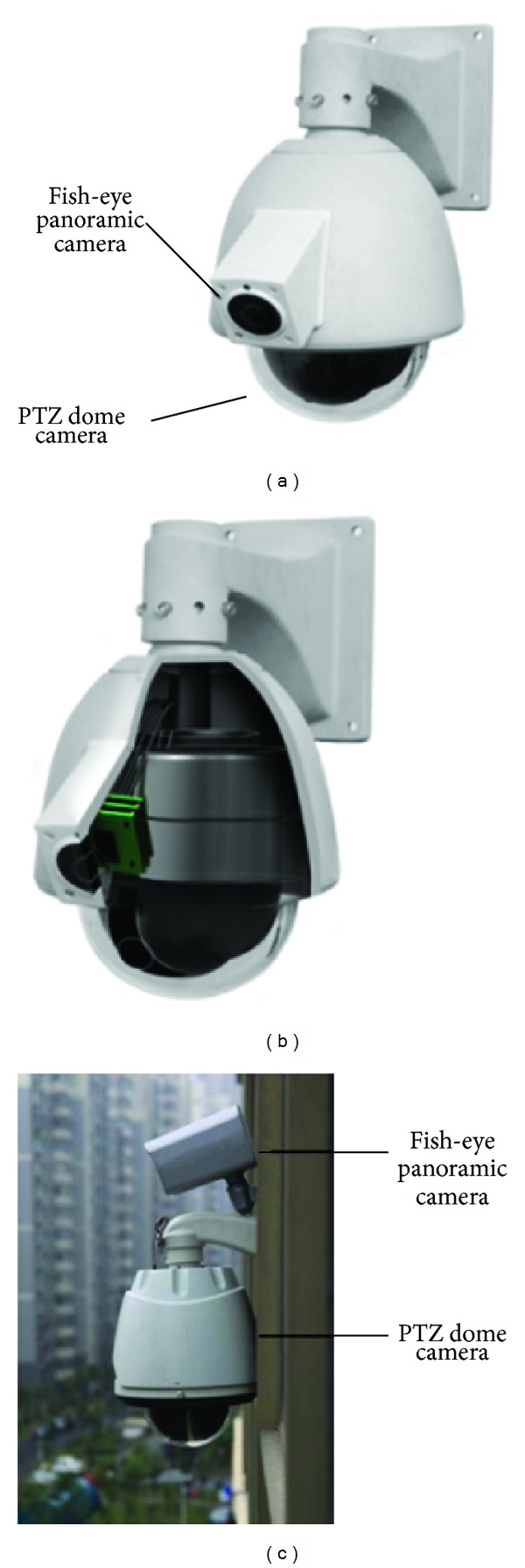
(a) The master-slave visual surveillance system. (b) The internal structure of the proposed system. (c) Most existing surveillance systems can be upgraded to the master-slave system by adding a fish-eye camera above the traditional PTZ camera, or adding a PTZ dome camera underneath the panoramic camera.

Figure 2(b) demonstrates the internal structure of the proposed system, where a panoramic camera acts as master camera and is mounted next above the traditional PTZ dome camera (Figure 2(a)). Upgrading from existing surveillance systems to a master-slave system is simple (shown in Figure 2(c)). This system can automatically direct a slave PTZ dome camera(s) to zoom into target areas of interest, in which details of object appearance are available at a higher resolution.

Different master-slave camera systems use various compositions of PTZ dome cameras and mixtures of other types of camera. Regardless of how the system is composed of camera types, the critical technique is to develop a suitable calibration algorithm for accurate interaction between the cameras. Through calibration, we hope that the target appointed by master camera can steer the slave camera to focus on the same position at the pixel level. The simplest and most direct way to precisely calibrate two cameras is to manually find every pixel in an image captured by one camera and correspond these with pixels in an image captured using another camera. Dense mapping such as this is impractical and it seriously limits the applicability. In practice, fewer points are required, which can be interpolated within some degree of accuracy. However, often the level of accuracy is not unacceptable. It has been shown experimentally that calibration based on 200 sample points may take several hours. Hence, a practical calibration method is required for master-slave camera systems.

**Figure 3:**
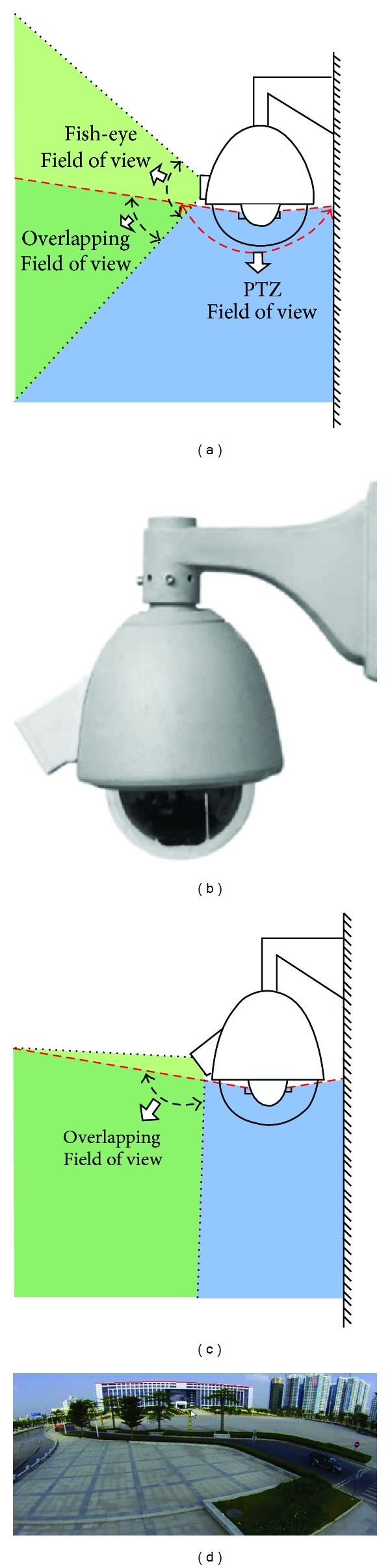
(a) If the fish-eye camera is mounted parallel to front, two cameras only have small overlapping FOV (b) The side view of master-slave visual surveillance system (c) The inclining angle ensures that the PTZ dome camera covers the FOV of fish-eye camera. (d) Image distortion appeared on the panoramic camera marked by the red curve line.

To ensure the panoramic camera captures the same scene content that the PTZ camera covers, the fish-eye camera is fixed inclining towards the gravity direction (Figures 3(a)–3(c)). However, this design brings serious image distortion in the horizontal direction (Figure 3(d)). So, an additional challenge for the proposed system is to find a suitable image correction to handle these distortions.

Our contributions over existing competing systems are twofold: (1) in terms of camera calibration, an efficient and accurate calibration method is proposed to accomplish the calibration between stationary and dynamic cameras. This method does not require specific camera setup or a particular grid pattern; (2) in terms of fish-eye distortion correction, our technique correctly handles the particular type of distortion introduced in fish-eye panoramic images. By adjusting the values of interrelated parameters, the extent of the distortion can be controlled. Moreover, the proposed algorithm can be applied to an embedded camera platform without any extra hardware resources due to its low computational cost.

The remainder of the paper is organized as follows.  Section 2 reviews related work.  Section 3 introduces the calibration method between panoramic and PTZ dome cameras.  Section 4 describes the proposed distortion correction algorithm. In Section 5, experiments are implemented and the experimental results are shown. Finally, Section 6 concludes this paper.

## 2. Related Work

As mentioned, the main challenge in the application of the proposed system is to actively control a PTZ dome camera to correctly focus on the same target in the panoramic scene. The precision of this interaction largely depends on the accuracy of the spatial calibration, which can be considered as the mapping between each of pixels in fish-eye panoramic image and the pan-tilt angles of PTZ dome camera. A practical calibration method should not only be efficient and effective but also need no particular system setup or human intervention. For the proposed system, it also has a problem of horizontal distortion when the master camera is mounted with an angle towards the gravity direction. Here we start with reviewing the calibration methods and approaches used in maser-slave camera system.

Current calibration methods can be divided into two main categories: geometry calibration and data fitting calibration. Geometry is a calibration method through single camera calibration and dual camera joint calibration to obtain the mapping relationship. However, it requires a priori knowledge about camera imaging model and geometric environment. Sato et al. [1] present an indoor monitoring system with multiple camera units, which includes panoramic camera and PTZ camera. They calculated the PTZ camera rotation angle corresponding to the point *P*(*P*_*x*_, *P*_*y*_) on the panoramic image using the following equation:

(1) $$\begin{array}{c} \theta_{P}=360\times \frac{P_{x}}{W+E_{x}},\\ \theta_{T}=\left(\left|OmniTop\right|+\left|OmniBottom\right|\right)\times \frac{P_{y}}{H+E_{y}}, \end{array}$$

where *E*_*x*_ and *E*_*y*_ are the distances between panoramic camera and PTZ camera in the horizontal and vertical direction respectively. *OmniTop* is the height from PTZ camera to the ground, and *OmniBottom* is the height from PTZ camera's bottom to the ground; *H* and *W* are the height and width of panoramic image. This simple method has large error because PTZ rotation angle is calculated through the position of point *P* in the panoramic image and relative shift of PTZ rotation angle. Scotti et al. [2] employed the master-slave camera in which the optical axis of catadioptric panoramic camera coincides with the horizontal axis of PTZ camera. They approximately assumed that the optical centers of two cameras overlap with each other due to the close installation. As the polar angle of the pixel in the panoramic image equals the corresponding horizontal rotation angle of PTZ camera, it simplifies the calculation greatly. It also reduces the error of the horizontal rotation angle which is brought by the change of the optical center with PTZ's movement. However, the method assumes that objects in the scene should be located on the same space ground. This hypothesis is invalid for most practical circumstance. The distance measurement between PTZ camera and the ground is required to be manually set, which also limits the installation procedure.

Geometry calibration generally needs to know two priories and satisfy a hypothesis, so the mapping relationship largely depends on both the accuracy of the priori and the validity of the hypothesis. While, the data fitting models the relationship between the panoramic coordinates and PTZ rotation angle by fitting the sample points. Both the camera imaging model and relative position of two cameras can be ignored, so this type of methods is more flexible. Hampapur et al. [3] triangulated a position by two or more calibrated cameras and determine the steering parameters for a third PTZ camera which is also calibrated. Chen et al. [4] proposed a versatile method for a variety of cameras. However, their method is at the cost of reducing the accuracy. They sampled the pixel coordinates (*x*, *y*) in the panoramic image and the correspondent PTZ rotation angle and hope to find the best fitting polynomial to describe their relationship. Tan [5] proposed a mapping method based on image piecewise fitting to obtain an improvement. Different polynomials were used according to the distortion degree in different areas. Nevertheless, the accuracy does not meet requirements. Senior et al. [6] used a master-slave camera that is composed of a fixed super wide-angled camera and a PTZ camera. They selected several sample points in the FOV of super wide-angled camera and determined nonsample point mapping relationship by a linear interpolation. Zhou et al. [7] selected a number of pixel locations in a static camera. For each pixel, manually move the slave camera to center the slave image and record the corresponding slave pan-tilt angles to obtain a lookup table. It links the static camera coordinates with the pan and tilt angles. Although their method is accurate enough to initialize the track of dynamic camera, it is time consuming and inconvenient. You et al. [8] employed a mosaic image created by snapshots of slave camera to estimate the relationship between static master camera plane and pan-tilt controls of slave camera. Compared with other approaches, this solution provides an efficient and automatic way to calibration of a master-slave system. Nevertheless, the mapping determined by a liner interpolation is inaccurate.

In terms of fish-eye panoramic image distortion correction, Devernay and Faugeras [9] assumed the presence of straight lines in the scene. Distortion parameters are sought which lead to lines being imaged as straight in the corrected image. Kannala and Brandt [10] proposed a novel calibration method for fish-eye lens cameras that was based on viewing a planar calibration pattern. This method was proven suitable for different kinds of omnidirectional cameras as well as for conventional cameras. Wang et al. [11] presented a new model of camera lens distortion that utilized two angular parameters and two linear parameters. These parameters were used to determine the transform from an ideal plane to real sensor array plane, which governs the lens distortion. Yu [12] proposed a lens geometric and photometric distortion correction method to obtain a high quality image. By using a simplified camera calibration technique, lens geometric coefficient can be estimated. Photometric distortion was corrected using a nonlinear model fitting of a proposed photometric distortion model function. Ying et al. [13] used spherical perspective projection model to calibrate the fish-eye lenses. Based on straight line spherical perspective projection constraint, the mapping between a fish-eye image and its corresponding spherical perspective image was determined. Once the mapping is obtained, the fish-eye lenses can be calibrated. Since orthographic spherical perspective projection was employed, these algorithms can only be applied to orthographic fish-eye cameras but not for equidistant fish-eye cameras [14, 15]. Li et al. [16] presented an embedded real-time fish-eye image distortion correction algorithm, which can be applied in an IP network camera. However, this algorithm only aimed to correct the distortion in the vertical direction. Moreover, methods that adapted the projection to content in the scene were also presented [17–19]. However, these methods require human intervention, and the corrected image has to be cropped.

Most previous distortion correction research focuses on constructing and calculating the internal reference model, which can express the mapping between the three-dimensional world and the two-dimensional image. Based on the internal reference model, the distorted image is mapped onto a three-dimensional spherical surface or parabolic surface. By using perspective projection, the distortion can be corrected. However, these methods aim to correct the distortion of conventional fish-eye panoramic images, and few methods have been proposed to correct the particular type of distortion in the fish-eye panoramic image captured by a master-slave camera. The proposed system draws inspiration from Midpoint Circle Algorithm (MCA) [16] and applies this algorithm to correct these distortions.

## 3. Calibration

Through a specific calibration, the target appointed by master camera can steer the slave camera to focus on the same position. To achieve this goal, the core technique is to determine the geometric relationship between the master camera image pixel coordinates and the pan-tilt angles of the slave camera.

### 3.1. Analysis of Coordinate Systems

The basis of calibration is to establish three coordinate systems: a panoramic coordinate system, a PTZ coordinate system, and a spherical coordinate system. As a master-slave camera is composed of a fish-eye panoramic camera and a PTZ dome camera, the panoramic coordinate system is a fish-eye coordinate system based on the fish-eye panoramic images, the PTZ coordinate system is a coordinate system based on PTZ camera taking pan angle and tilt angle as parameters, and the spherical coordinate system is an auxiliary coordinate system transforming from the panoramic coordinate system to the PTZ coordinate system.

**Figure 4:**
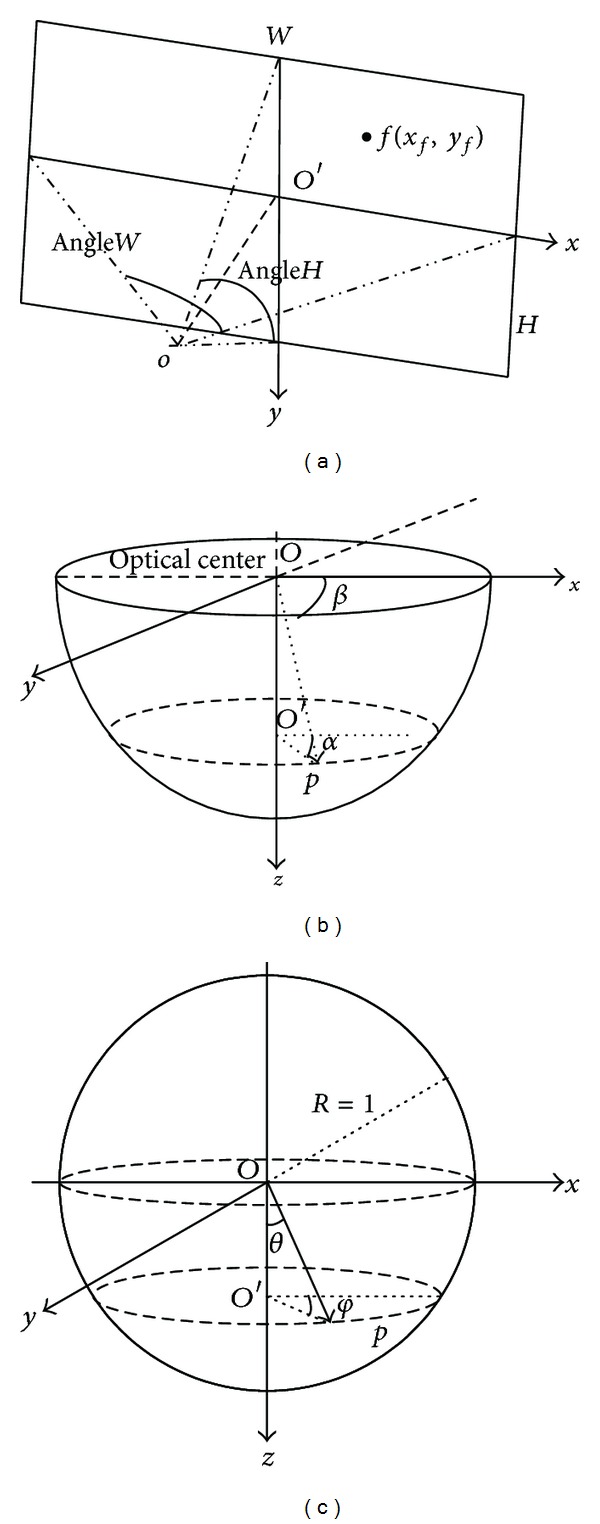
(a) Panoramic coordinate system, (b) PTZ camera coordinate system, and (c) PTZ image coordinate system.

Figure 4(a) illustrates the panoramic coordinate system. The *x*-axis and the *y*-axis represent the horizontal and vertical directions, respectively. The optical center is *O*, and *OO*′ is the optical axis. *W* and *H* are the width and height of the panoramic image respectively. *AngleW* and *AngleH* are, the horizontal and vertical angle of view of the panoramic image, respectively. The focal length of fish-eye panoramic camera *C*_*f*_ can be calculated by *W*/*AngleW*.

Here we set (*x*_*f*_, *y*_*f*_) as the pixel positions in the panoramic image. As shown in Figure 4(b), the PTZ coordinate system contains two parameters. *p* is a point on the surface of sphere. *α* represents the pan angle between *O*_*p*_′ and positive *x*-axis. It increases in the anticlockwise direction viewing from positive *z*-axis, which ranges from 0° to 359°. *β* is the tilt angle that ranges from 0° to 89°. It is the angle between *O*_*p*_ and *XOY *plane which increases in the clockwise direction viewing from positive *x*-axis. The PTZ coordinate system is defined within a hemisphere on the *XOY* plane with the *z*-axis pointing downwards.

Figure 4(c) illustrates the unit spherical coordinate system. Starting point *O* and every axis correspond to the PTZ dome coordinate system. The coordinate of point *p* is denoted by (*x*_*s*_, *y*_*s*_, *z*_*s*_). *φ* is the angle between *O*_*p*_′ and positive *x*-axis which increases in the anticlockwise direction viewing from positive *z*-axis and ranges from 0° to 359°. *θ* is the angle between *O*_*p*_ and positive *z*-axis which increases in the anticlockwise direction viewing from positive *x*-axis and ranges from 0° to 89°.

### 3.2. Transformation between Coordinate Systems

When the transformation from the panoramic coordinate system to the PTZ coordinate system is obtained, we can determine the mapping relationship between each of pixels in the image captured by the fish-eye camera and image captured by the PTZ camera. However, the transformation cannot be calculated in a single step directly. We need to use spherical coordinate system to link with these two coordinate systems. The process is completed in three steps. Firstly, assume (*x*_*f*_, *y*_*f*_, 1) to be any pixel with homogeneous coordinates in the panoramic coordinate system with a corresponding spherical homogeneous coordinate (*x*_*Ps*_, *y*_*Ps*_, *z*_*Ps*_, 1). Secondly, set the mapped homogeneous coordinate to be (*x*_*P*_, *y*_*P*_, *z*_*P*_, 1). In the PTZ coordinate system, its corresponding spherical homogeneous coordinate is (*x*_*Hs*_, *y*_*Hs*_, *z*_*Hs*_, 1). Last, establishing a mapping relationship between (*x*_*Ps*_, *y*_*Ps*_, *z*_*Ps*_, 1) and (*x*_*Hs*_, *y*_*Hs*_, *z*_*Hs*_, 1) as follows:

(2) $$\begin{array}{c} \left(x_{Hs},y_{Hs},z_{Hs},1\right)=\left(x_{Ps},y_{Ps},z_{Ps},1\right)\times T_{PH}. \end{array}$$

*T*_*PH*_ is a 4 × 4 matrix which represents the transformation from a panoramic spherical coordinate to PTZ dome spherical coordinate. Before the transformation, there are two constraints for the master-slave camera system. Firstly, the camera's optical axis is perpendicular to the image plane and point of intersection is at the center of the image plane. Secondly, the fish-eye panoramic camera's optical center and the PTZ dome camera's optical center are in the same vertical plane. If above two constraints are not satisfied, great errors may result. The mapping *T* is obtained through a process of transformation among coordinates:

(1) Transformation from panoramic coordinate system to spherical coordinate system.

As shown in Figure 3(a), we can obtain the distance *R*_*f*_ between point *p* and the center. Based on the fish-eye imaging model, the radical angle of point *p* off-center is

(3) $$\begin{array}{c} \theta =\frac{R_{f}}{C_{f}}. \end{array}$$

According to *R*_*f*_ and *θ*, the *z*-axis of the point *p* can be calculated. So the transformation is

(4) $$\begin{array}{c} x_{Ps}=Sx_{f},\\ y_{Ps}=Sy_{f},\\ z_{Ps}=S\frac{R_{f}}{\tan\left(R_{f}/c_{f}\right)}. \end{array}$$

*S* is the normalization constant to ensure $\sqrt{x_{Ps}^{2}+y_{Ps}^{2}+z_{Ps}^{2}}=1$.

(2) Transformation from PTZ coordinate system to spherical coordinate system.

As PTZ dome camera model is a hemisphere model, the PTZ coordinate system is identical with the spherical coordinate system. The transformation can be written as

(5) $$\begin{array}{c} x_{Hs}=\cos\varphi \sin\theta ,\\ y_{Hs}=\sin\varphi \sin\theta ,\\ z_{Hs}=\cos\theta . \end{array}$$

According to the transformation using the two steps described above, the final transformation can be obtained.

### 3.3. Calibration Theory between Master and Slave Camera

**Figure 5:**
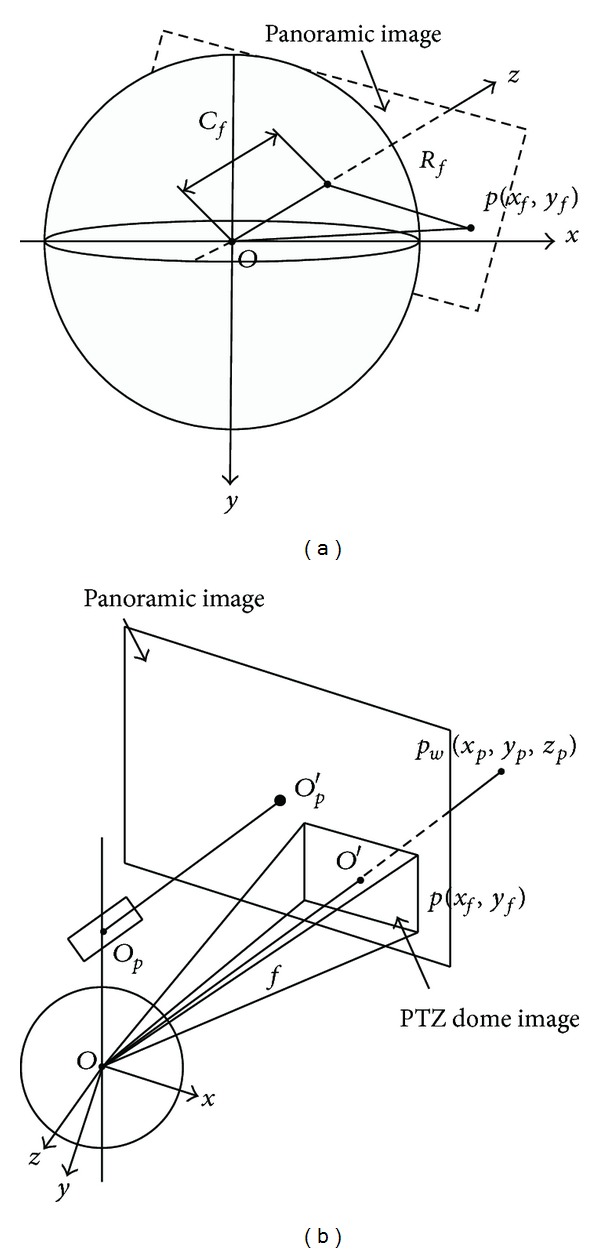
(a) PTZ image coordinate system imaging and (b) PTZ camera's pinhole.

(*x*_*f*_, *y*_*f*_) are the coordinates of pixel *p* in the panoramic coordinate system. (*x*_*p*_, *y*_*p*_, *z*_*p*_) are the coordinates of the corresponding point in the PTZ coordinate system. The mapping *T* can be determined by finding a matrix that makes the optical centers of the two cameras coincide with each other. Namely, a panoramic image can be considered as a large PTZ dome image. A PTZ dome camera satisfies the theory of pinhole imaging. According to Tsai [20], the theory of pinhole camera imaging is illustrated in Figure 5(b), in which $s\tilde{m}=PX_{c}$.

In the proposed system, a fish-eye panoramic camera is mounted above a PTZ dome camera and their optical centers are on the same vertical plane. Here *O*_*p*_ is the optical center of panoramic camera and *O*_*p*_′ is its principal point. *O*_*p*_ is the optical center of PTZ dome camera and *O*′ is its principle point. *p* is a point in the panoramic coordinate system and *p*_*w*_ is the corresponding object point in the world coordinate system. We project the PTZ dome image on the panoramic image. According to pinhole imaging theory, if the PTZ dome camera shoots at *p*_*w*_, *O*′ will coincide with *p*, which is the result we hope to obtain. In this work, the mapping *T* is calculated through a process of selecting sampling points. According to the algorithm presented by Zhang [21], it has to select at least 3 PTZ dome image to solve the transformation matrix.

### 3.4. Feature Points Matching

This step requires a method to detect and match visual feature that is robust to scale, rotation, viewpoint, and lightning. The Scale Invariant Feature Transform (SIFT) [22] exhibits great performance under these requirements. In this work, we employ SIFT to detect feature points in both the panoramic image and the PTZ dome image. Consider that {*P*_*Hi*_^*j*^} = {(*X*_*Hi*_^*j*^, *Y*_*Hi*_^*j*^, *Z*_*Hi*_^*j*^)}^*T*^(*i* = 1,…6; *j* = 1,…*n*, *n* ≥ 6) is the *j*_th_ feature point in the spherical coordinate of the *i*_th_ PTZ dome image. Consider that {(*P*_*Pi*_^*j*^)} = {(*X*_*Pi*_^*j*^, *Y*_*Pi*_^*j*^, *Z*_*Pi*_^*j*^)}^*T*^(*i* = 1,…6; *j* = 1,…*n*, *n* ≥ 6) is the panoramic spherical coordinate that corresponds to {*P*_*Hi*_^*j*^}. *A*_*Hi*_ is the feature point matrix in the *i*th PTZ dome image. *A*_*Pi*_ is the matching feature point matrix in the panoramic image:

(6) $$\begin{array}{c} T_{i}=A_{Pi}\times {\left(A_{Hi}^{T}A_{Hi}\right)}^{-1}A_{Hi}^{T}, \end{array}$$

where *A*_*Hi*_ = [*P*_*Hi*_^1^, *P*_*Hi*_^2^,…, *P*_*Hi*_^*m*^] and *A*_*Pi*_ = [*P*_*Pi*_^1^, *P*_*Pi*_^2^,…, *P*_*Pi*_^*m*^]. *P*_*Hi*_^0^ is the center point spherical coordinate of the *i*th PTZ dome image. *P*_*Pi*_^0^ is the panoramic spherical coordinate that corresponds to *P*_*Pi*_^0^:

(7) $$\begin{array}{c} P_{Pi}^{0}=T_{i}\times P_{Hi}^{0}\;\left(i=1,\ldots n\right). \end{array}$$

Here *A*_*H*_ is the center point spherical coordinate matrix in the PTZ dome image. *A*_*P*_ is the matching point matrix in the panoramic image:

(8) $$\begin{array}{c} T_{PH}=A_{P}\times {\left(A_{H}^{T}A_{H}\right)}^{-1}A_{H}, \end{array}$$

where *A*_*H*_ = [*P*_*H*1_^0^, *P*_*H*2_^0^,…, *P*_*Hn*_^0^] and *A*_*P*_ = [*P*_*P*1_^0^, *P*_*P*2_^0^,…, *P*_*Pn*_^0^]. The calibration can be considered as calculating the transformation matrix. As a result, for given any pixel, we can calculate the corresponding rotation angle. The feature points matching step is employed to solve the unknown in the transformation matrix *T*_*PH*_.

## 4. Distortion Correction

As mentioned by Strand and Hayman [23], a straight line in world coordinates can be projected to a corresponding circle on the fish-eye image plane, which means that the mapping process can be calculated directly using MCA [16]. The proposed distortion correction method can be divided into two parts. Firstly, the coordinate mapping is calculated based on MCA between a column of the first corrected image and the arc line of the original fish-eye panoramic image in the vertical direction, which is named Vertical Correction Image (VCI) in this work. Secondly, following the same principle, the coordinate mapping between a row of the second corrected image and the arc line image of VCI in horizontal direction is calculated, which is named Horizontal Vertical Correction Image (HVCI) here.

**Figure 6:**
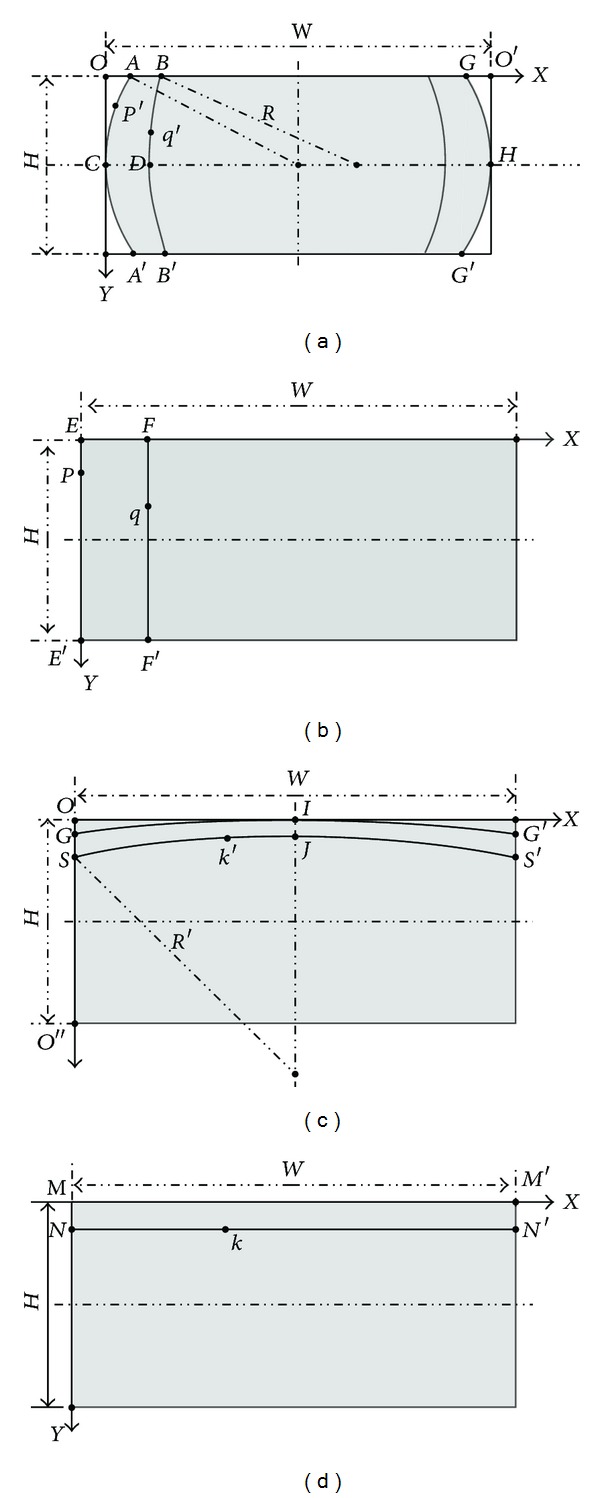
(a) The structure of captured fish-eye panoramic image in vertical direction. (b) VCI in vertical direction. (c) VCI in horizontal direction. (d) HVCI in horizontal direction.

Figures 6(a) and 6(b) demonstrate the structure of captured fish-eye panoramic image and VCI in vertical direction, whose width and height both are *W* and *H*. Figures 6(c) and 6(d) show the VCI and HVCI in the horizontal direction.

For vertical distortion correction, the first column *EE*′ in VCI corresponds to the first arc line *ACA*′ in the fish-eye panoramic image. For an arbitrary point *P* on *EE*′, there is a corresponding point *P*′ on *I*. Since the distortion is corrected only in the vertical direction, ordinate value of point *P* is the same with its corresponding point *P*′. In fish-eye panoramic image, *OA* does not project to VCI, so its length *L* is called Vertical Distortion Redundancy Length (VDRL).

For an arbitrary column *FF*′ in VCI, assume that its corresponding arc line image in the fish-eye panoramic image is *BDB*′. The point *F*(*X*_*f*_, *Y*_*f*_) on *FF*′ has its corresponding point *B*(*X*_*b*_, *Y*_*b*_) on *BDB*′. Based on MCA, for an arbitrary point *q*(*X*_*q*_, *Y*_*q*_) on *FF*′, assume that its corresponding point on *BDB*′ is *q*′(*X*_0_, *Y*_0_). Thus the corresponding point position can be calculated as follows:

(9) X0=λ2+δ2−Xf22(δ−Xf)−Xf+Xq −λ2+δ2−Xf22(δ−Xf)−Xf2−(λ−Y0)2 (Xq≤W2),X0=λ2+(W−δ)2−(W−Xf)22(Xf−δ)−ω+Xq+λ2+(W−δ)2−(W−Xf)22(Xf−δ)−ω2−(λ−Y0)2(W2<Xq≤W),Y0=YK,

where *λ* = *H*/2,  *δ* = *L* + *X*_*f*_((*W* − 2*L*)/*W*), and *ω* = *H* − *Y*_*k*_. The extent of the distortion correction can be controlled by the value of VDRL.

The distortion correction in the horizontal direction follows the same logic. In HVCI, an arbitrary row has its corresponding arc line in the VCI, such as the first row line image *MM*′ and its corresponding first arc line *GIG*′. With VDRL in fish-eye panoramic image, there also is Horizontal Distortion Redundancy Length (HDRL) *L*′ in the VCI, which is the length of |*OG*|. Because the distortion is corrected only in horizontal direction, the abscissa value of a point in HVCI is the same as its corresponding point in VCI. For an arbitrary row line *NN*′ in HVCI, assume that its corresponding arc line in VCI is *SJS*′. A point *k*(*X*_*k*_, *Y*_*k*_) on *NN*′ has its corresponding point *k*′(*X*_1_, *Y*_1_) on *SJS*′. The coordinate values of point *k*′ can be derived as follows:

(10) Y1=λ2+δ2−Yk22(δ−Yk)−(λ2+δ2−Yk22(δ−Yk)−Yk)2−(λ−X1)2(Yk≤H2),Y1=λ2+(H−δ)2−(H−Yk)22(Y−δ)−H+2Yk +(λ2+(H−δ)2−(H−Yk)22(Y−δ)−(H−Yk))2−(λ−X1)2(H2<Yk≤H),X1=Xk,

where *λ* = *W*/2 and *δ* = *L*′ + *Y*_*k*_((*H* − 2*L*′)/*H*). By traversing the HVCI, all positions can be mapped to corresponding points in VCI. Thus, the distortion in the horizontal direction can be corrected, and the extent of distortion correction can also be controlled by adjusting the value of HDRL.

After correcting the distortion in the vertical and horizontal directions, the distortion in the fish-eye panoramic image captured by master-slave camera can be corrected effectively and efficiently.

## 5. Experiments and Results

With regard to the time consuming of the proposed calibration method. The process can be considered as two steps: image subtracting and image matching. Capturing one image takes 2 seconds roughly. For one pair of images, image matching procedure takes about 5 seconds. In this circumstance, the entire process only takes 50 seconds. Compared with the traditional manual calibration which normally consumes several hours, the proposed automatic calibration algorithm increases the efficiency dramatically.

**Figure 7:**
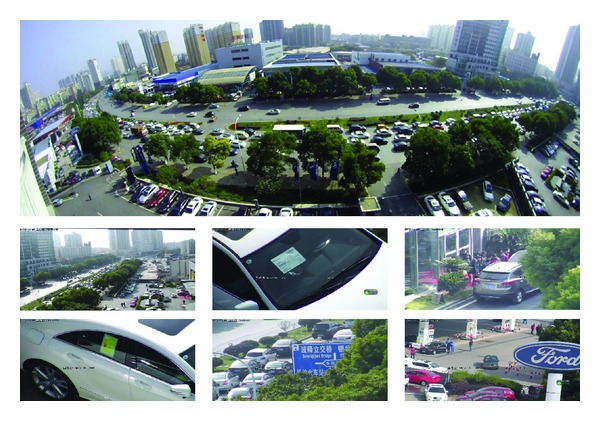
Experimental result after the calibration. Several areas are selected in the panoramic image as focus of interest, and corresponding multiscaled image is captured by PTZ dome camera.

For the accuracy evaluation, Figure 7 demonstrates the images captured by the proposed master-slave camera. Here we focus on four areas of the panoramic image which includes distinguished features.

**Figure 8:**
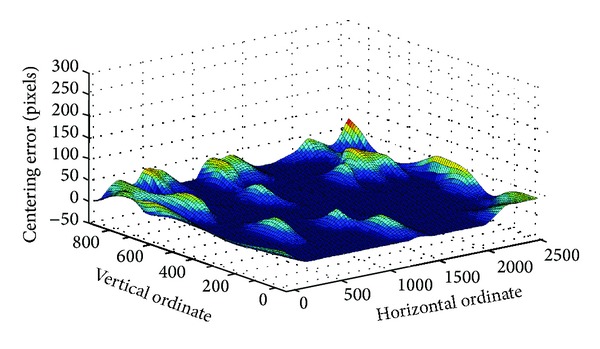
Mean error between requested and the ground truth pixel.

Figure 8 shows the mean error between requested pixel (*p*_*x*_, *p*_*y*_) and the ground truth pixel (*p*_*x*_′, *p*_*y*_′) to evaluate the distribution of error.

In Figure 8, the dark blue area represents 0~35 pixels, the green area represents 35~70 pixels, and the red area represents 70~105 pixels. In this work, the size of the PTZ dome image is 1920 ∗ 1080 pixels. It shows that error can be under-controlled within 1° over 95% of the scene. However, three main factors could influence the accuracy during the calibration procedure. Firstly, the PTZ dome camera only has accuracy of 1° other than 0.1°. Under this condition, 3.5 pixels is equivalent to 1° which bring the result that the centering error greatly reduces. Secondly, the selection of the PTZ dome images' position. Once the chosen 6 PTZ dome images are in uniform distribution in the panoramic image, the red area would not appear in Figure 8. Last but not least, it is the feature point matching process and the numbers of match points directly influence the result of calibration.

**Figure 9:**
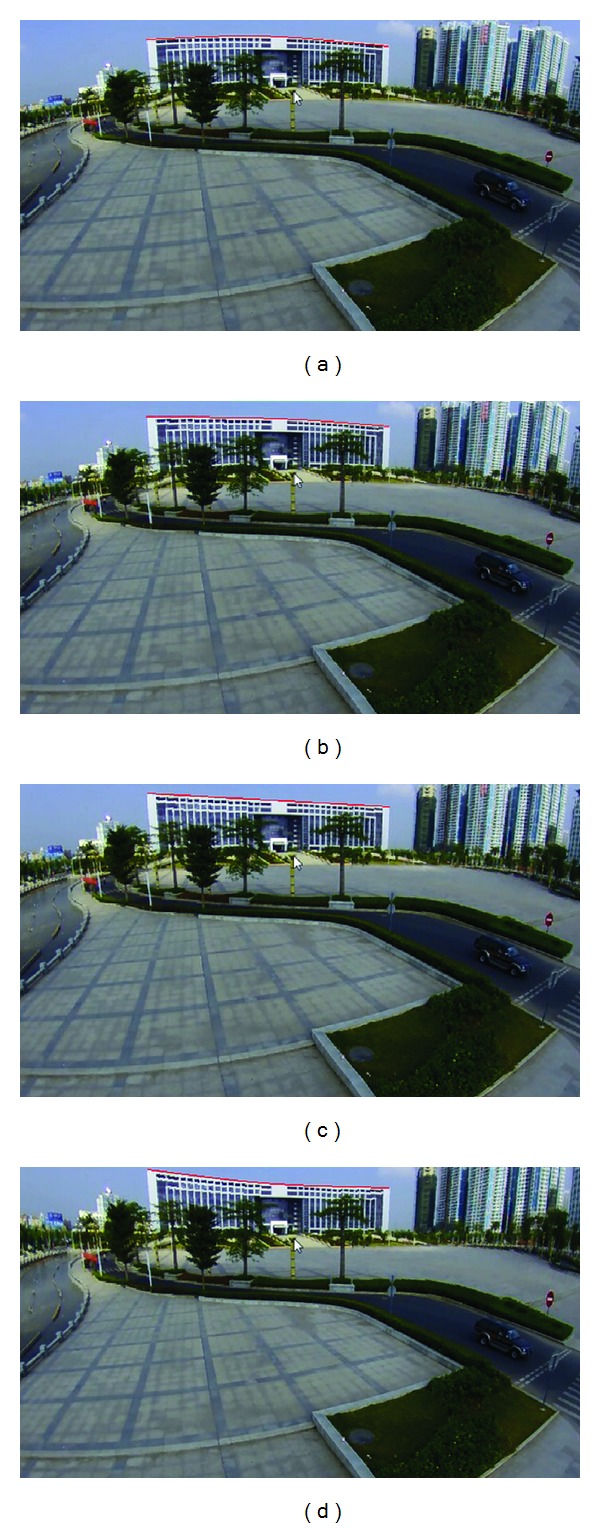
Different images after distortion correction with different Horizontal Distortion Redundancy Length (HDRL) values. (a)~(d) show the corrected images when HDRL values are 60, 90, 120, and 140, respectively.

Figure 9 shows the images after distortion correction with different HDRL values, and the correction results are marked by red lines to offer a more direct expression. The distortion in Figure 9(a) is corrected in a small scale without any obvious noticeability. Although the distortion correction result in Figure 9(b) is close to the realistic scene, it is still away from being ideal, while in Figure 9(c), the HDRL value is 120 in which the distortion in horizontal direction obtains a desirable correction effect. When the HDRL value is 140 (Figure 9(d)), the setting is obviously overdone and causes overdistortion. It is worth mentioning that the HDRL needs to be tested to obtain a relatively ideal value since it differs in different panoramic images.

Table 1 shows the comparison of time consumption on dealing with different resolution images with the algorithm proposed by Ying et al. [13]. The comparison was conducted with single-threaded implementation on 3.20 GHz Intel Core i5-3470 CPU and 4.00 GB RAM computer by using Microsoft Visual Studio 2008 software. The result shows that the proposed algorithm has much less time consumption than Ying's algorithm under different resolutions. Moreover, the calculation resource consumption between Ying's algorithm and the proposed algorithm is also listed, which includes the utilization of CPU and memory. As represented, less resource is required by the proposed correction method which enables the proposed surveillance system to achieve real-time performance.

## 6. Conclusion and Discussion

A master-slave camera system that is composed of panoramic and PTZ dome cameras is proposed for stationary and dynamic visual surveillance. A panoramic camera observes a scene with a large field of view, and PTZ dome cameras simultaneously capture high-resolution images with multiscale information. It can roughly cover 2-square-kilometer area with one camera, especially suitable for the large area surveillance such as squares and stadiums. More specifically, we present a calibration method for obtaining the mapping relations between master camera and slave camera. The availability and accuracy of the method are validated by the experiments shown in this paper. Additionally, we propose a correction approach to correct the particular type of distortion in fish-eye panoramic image captured by this camera system. It has been applied on embedded camera platform without any extra hardware resources due to its low computational cost. In order to achieve the more precise interaction, future work would consider a calibration method based on panoramic image mosaic to obtain the pixel level mapping relation between the fish-eye image and the PTZ camera's motion parameters.

## Figures and Tables

**Table 1:** Comparison of time and resource consumption.

Image resolution	Ying's algorithm [13]			Our algorithm		
	Time (ms)	CPU (%)	Memory (Kb)	Time (ms)	CPU (%)	Memory (Kb)
640 × 480	416	15	3284	30	1	1516
1280 × 720	1371	20	9524	212	4	1992
1920 × 1080	3125	20	19604	274	6	2336
2560 × 896	3605	20	25560	419	14	2692
